# Family Income Mediates the Effect of Parental Education on Adolescents’ Hippocampus Activation During an N-Back Memory Task

**DOI:** 10.3390/brainsci10080520

**Published:** 2020-08-05

**Authors:** Shervin Assari, Shanika Boyce, Mohsen Bazargan, Cleopatra H. Caldwell

**Affiliations:** 1 Department of Family Medicine, Charles R Drew University of Medicine and Science, Los Angeles, CA 90059, USA; Mohsenbazargan@cdrewu.edu; 2 Department of Pediatrics, Charles R Drew University of Medicine and Science, Los Angeles, CA 90059, USA; ShanikaBoyce@cdrewu.edu; 3 Department of Family Medicine, University of California Los Angeles, Los Angeles, CA 90095, USA; 4 Department of Health Behavior and Health Education, School of Public Health, University of Michigan, Ann Arbor, MI 48104, USA; cleoc@umich.edu; 5 Center for Research on Ethnicity, Culture, and Health (CRECH), School of Public Health, University of Michigan, Ann Arbor, MI 48104, USA

**Keywords:** socioeconomic factors, hippocampus, adolescents, brain development, fMRI

## Abstract

*Introduction:* Hippocampus, a medial temporal lobe structure, has significant implications in memory formation and learning. Although hippocampus activity is believed to be affected by socioeconomic status (SES), limited knowledge exists on which SES indicators influence hippocampus function. *Purpose:* This study explored the separate and combined effects of three SES indicators, namely parental education, family income, and neighborhood income, on adolescents’ hippocampus activation during an N-Back memory task. As some of the effects of parental education may be through income, we also tested if the effect of parental education on hippocampus activation during our N-Back memory task is mediated by family or neighborhood income. *Methods:* The Adolescent Brain Cognitive Development (ABCD) study is a national multi-center investigation of American adolescents’ brain development. Functional magnetic resonance imaging (fMRI) data of a total sample of 3067 9–10-year-old adolescents were used. The primary outcome was left- hippocampus activation during the N-Back memory task (mean beta weight for N-Back run 1 2 back versus 0 back contrast in left hippocampus). The independent variable was parental education. Family income and neighborhood income were two possible mediators. Age, sex, and marital status were the covariates. To test mediation, we used hierarchical linear regression models first without and then with our mediators. Full mediation was defined according to Kenny. The Sobel test was used to confirm statistical mediation. *Results:* In the absence of family and neighborhood income in the model, higher parental educational attainment was associated with lower level of left hippocampus activation during the N-Back memory task. This effect was significant while age, sex, and marital status were controlled. The association between parental educational attainment and hippocampus activation during the N-Back memory task was no more significant when we controlled for family and neighborhood income. Instead, family income was associated with hippocampus activation during the N-Back memory task. These findings suggested that family income fully mediates the effect of parental educational attainment on left hippocampus activation during the N-Back memory task. *Conclusions:* The effect of parental educational attainment on adolescents’ hippocampus activation during an N-Back memory task is fully explained by family income. That means low family income is why adolescents with low-educated parents show highlighted hippocampus activation during an N-Back memory task. Given the central role of the hippocampus in learning and memory and as income is a modifiable factor by tax and economic policies, income-redistribution policies, fair taxation, and higher minimum wage may have implications for promotion of adolescent equality and social justice. There is a need to focus on family-level economic needs across all levels of neighborhood income.

## 1. Introduction

Low socioeconomic status (SES) is a risk factor of poor adolescents’ brain development [1]. Adolescents from low SES backgrounds show worse brain function than those from high SES backgrounds, a difference that can be explained by poverty [2,3,4]. Due to lower brain function, adolescents from low SES families are at an increased risk of psychopathology [5], emotion dysregulation [6,7], behavioral problems [8,9,10], poor school performance [11], and attention deficit and hyperactivity disorder [12,13,14,15]. Worse brain function may also explain why low SES adolescents are at an increased risk of antisocial behaviors [16], aggression [17], early sexual initiation [18], as well as use of tobacco [19,20], alcohol [21,22], and drugs [23].

Although various brain regions and structures may carry some of the effects of low SES on brain function, the role of the hippocampus is essential [24,25,26,27,28]. Hippocampus, a medial temporal lobe structure, with major implications for positive social relations, emotion regulation, memory formation [29,30,31,32,33], and learning [34,35,36,37,38], has shown a high degree of sensitivity to low SES [24,25,26,27,28]. Altered hippocampus function may also have a role in problem behaviors [39,40], aggression [41,42,43], psychopathology [12,44], post-traumatic stress disorder (PTSD) [45,46,47,48,49,50,51], depression [52,53,54], and drug use [55,56,57,58,59]. Thus, altered hippocampus function may be one of the mediators of the effect of low family SES on several associated behaviors [12,25,27,35,60,61,62,63,64,65,66].

Although a considerable body of the literature has shown a link between SES and adolescents’ brain development, most of this research has focused on brain structures other than the hippocampus [2,67,68,69]. That means most of the existing knowledge is on the effects of SES and associated stress and adversities on the amygdala structure and function [2,67,68,69]. For example, Javanbakht et al. documented the effects of low-income and associated childhood adversities on the amygdala’s over-reactivity to negative stimuli [2,68,69]. Less is known, however, on the effects of SES and associated stress on the hippocampus [26,27,28,30,35,60,64,70].

Detailed and nuanced knowledge regarding the effects of various SES indicators on brain development may enable us to reduce the economic and social inequalities in child brain development. This is important because SES effects are non-specific to any particular domain and can be seen across outcomes such as school performance [71], mental health [72], emotion regulation [73,74], aggression [75], and substance use [72,76]. An enhanced understanding of the SES indicators that determine adolescents’ brain function is essential for breaking the vicious cycle between low SES and poor emotional, behavioral, and developmental domains.

Some studies have suggested that parental education [77,78,79], family income [67], and neighborhood-level income [66,80,81] may all relevant determinants of brain function. However, it is unknown which SES indicator has a more salient role than others. According to some studies, family income is particularly consequential in terms of brain development of the children, particularly those who live in most disadvantaged sections of the society [67]. For children who lived under poverty, a small increase in income was associated with a relatively significant increase in the brain’s surface area. Among high SES children, however, a similar incremental change in family SES (i.e., income) was associated with relatively fewer changes in the brain’s surface area [67].

There is also a need to decompose the effects of SES indicators such as parental education, family income, and neighborhood income on brain health. Although these SES indicators have close overlap [82], income may be one reason why parental education impacts brain development. In this view, income, not the parental education, is the final SES indicator that shapes the adolescents’ development [83]. From another side, parental education and income may have different effects on the brain development of adolescents [67]. However, not much research has generated knowledge on which SES indicator most contributes to outcomes. Ross and Mirowsky [84,85] and others [86,87,88,89,90] have provided some theoretical and empirical evidence suggesting that income partially explains the effects of education on health [91,92]. These are particularly important because it might be easier to change income, in a short time period, than parental education. Thus, if income is the final solution, policymakers may be able to prevent social inequalities in adolescents’ brain development via implementing policies that raise the pay of low-income people [93,94].

### Aims

To understand the social patterning of hippocampus activation during an N-Back memory task, we used the Adolescent Brain Cognitive Development (ABCD) study data to investigate the effects of three SES indicators (i.e., parental education, household income, and neighborhood income) on hippocampus activation during an N-Back memory task. We tested separate and additive effects of parental education and family and neighborhood income as three leading SES indicators. We also tested the mediating effect of family and neighborhood income as potential mechanisms (mediator) for the effect of parental educational attainment on hippocampus activation during an N-Back memory task. Finally, we validated hippocampus activation during an N-Back memory task by using the Child Behavior Checklist (CBCL), also called the Achenbach System of Empirically Based Assessment (ASEBA), one of the most commonly used measures of adolescent behavior, social, and emotional problems [95,96,97,98,99,100]. As hippocampus function is linked to problem behaviors [39,40], aggression [41,42,43], psychopathology [12,44], anxiety [45,46,47,48,49,50,51], depression [52,53,54], and drug use [55,56,57,58,59], we expected some associations between CBCL domains and CBCL total score and the hippocampus function during the memory task.

## 2. Methods

### 2.1. Design and Settings

This is a secondary analysis with a cross-sectional design. This study borrowed data from the ABCD study [101,102,103,104,105]. With a cross-sectional design, wave 1 data of the ABCD study were used. ABCD is a national, state-of-the-art brain imaging study of adolescents’ brain development [101,106].

### 2.2. Ethical Aspect

The ABCD study protocol is approved by the Institutional Review Board (IRB) at the University of California, San Diego (UCSD). A few other sites were obtaining local IRB approval. All adolescents signed assent. Adults signed informed consent [106]. Given out use of de-identified data, our study was non-human subject research.

### 2.3. Participants and Sampling

The ABCD participants were selected across multiple US states. The recruitment was predominantly through school systems. School selection was informed by sex, race, ethnicity, socioeconomic status, and urbanicity. Detailed information of the ABCD sampling is published before [107]. The current analysis used an analytical sample of 3067 non-Hispanic White or Black adolescents. Inclusion in this analysis was based on complete data on the variables race, ethnicity, SES, and left hippocampus activation during an N-Back memory task.

### 2.4. Functional MRI and Image Acquisition

T1 weighted structural and T2 weighted functional magnetic resonance imaging (fMRI) images were taken using a 3 tesla (T) Siemens Prisma, General Electric 750, and Phillips multi-channel coiled scanners, all capable of multiband echo-planar imaging (EPI) acquisitions [102]. A localizer is implemented at the beginning of each scan, followed by the T1 weighted structural image acquisition. Functional T2 weighted scans are then acquired throughout the tasks. Three separate tasks are required of all participants, which include the monetary incentive delay task (not used in the proposed study), Stop Signal task (SST), and EN-Back; the order in which these paradigms are introduced is randomized between subjects. Structural T1 weighted scan sequences are optimized for cortical and subcortical segmentation via magnetization-prepared rapid acquisition gradient-echo.

### 2.5. ABCD Study Neuroimaging Data

Large-scale multimodal data acquisition has allowed the ABCD study to collect an unprecedented imaging data set on a large number of adolescents across 21 data acquisition sites in the US. This large number of participants increases the statistical power of the data analysis in this cohort. This is especially important given the costs of imaging. In addition, low statistical power is among the most limiting common weaknesses of MRI studies (especially functional MRI; fMRI). This is especially important for examination of the impact of sex in the observed differences, as it will even further reduce power in a smaller sample size, as well as longitudinal examination of changes in developing brain structure and function. ABCD structural and functional data is already processed and the tabulated regions of interest (ROI) data are available through the NIMH Data Archive (NDA). ABCD imaging data include T1-weghted, T2-weighted, structural MRI (sMRI), fMRI (both resting-state and task based), and diffusion MRI. The fMRI tasks include monetary delayed incentive task, stop signal task, and emotional N back task (EN-back). Over 78% of the participants from the ABCD data release 1.1 completed the imaging protocols [108]. The fMRI tasks of interest for our proposed study include the N-Back task. Details of fMRI data processing can be seen in Hagler et al., 2018 [108].

### 2.6. N-Back Task

The N-Back model includes some predictors for various types of the stimulus (emotional face and place) across N-Back conditions (i.e., 2-back) plus fixation. Linear contrasts are obtained for each memory load and each stimulus type versus fixation. Similarly, linear contrasts are obtained for “2-back” versus. “0-back across” stimulus types [2]. Region of interest (ROI) in the current analysis was left hippocampus activity during the N-Back task. This ROI was selected based on an extensive literature reviewed in the introduction and discussion. Derived from data sets that had already defined the ROIs covering the entire regions, ABCD has left hippocampus ROI based on Gordon and colleagues in 2016 [109]. Appendix A provides the distribution of hippocampus activation during the N-Back memory test. As his histogram shows, our outcome has a near to normal distribution shape. For the N-Back task, we choose 2 versus 0 because it is a more difficult task than 1 versus 0. Only correct trials used. So, we could differentiate individuals who had good and poor memory function during the N-Back task.

### 2.7. Variables

Variables in this study included demographic factors (age and sex), SES indicators (parental education, family income, and neighborhood income), and brain function (mean beta weight for left- hippocampus during an N-Back memory task (run 1 2 back versus 0 back contrast). Details of the procedures for tasks as well as fMRIs are explained here [102].

#### 2.7.1. Outcome

The outcomes were the hippocampus function measured during an N-Back memory task. We operationalized this variable as mean beta weight for N-Back run 1 2 back versus 0 back contrast in the subcortical automatic segmentation ASEG regions of interest (ASEG ROI) in left hippocampus. We selected hippocampus because it is shown to be the leading brain function indicator of social adversities associated with poverty, economic hardship, and trauma [30,63,64,65,66,70,110,111,112].

#### 2.7.2. Independent (Predictor) Variable

*Parental Educational Attainment.* Participants reported their years of schooling. This variable was operationalized as a continuous (interval) variable ranging from 0 for no formal education to 21 doctoral degrees.

#### 2.7.3. Mediator

*Family income.* Family income was a 1–10 interval measure where a higher score indicated higher income. The total combined family income in the past 12 months was asked. Responses were 1 = less than $5000; 2 = $5000; 3 = $12,000; 4 = $16,000; 5 = $25,000; 6 = $35,000; 7 = $50,000; 8 = $75,000; 9 = $100,000; 10 = $200,000. Appendix B presents the summary of the income variable.

*Neighborhood income*. Derived from the ABCD residential history file, we used the neighborhood’s median family income (for the first residential address as neighborhood SES). This is a component of the area deprivation index (ADI) developed by the Health Resources & Services Administration (HRSA). This is based on the neighborhood income of the county-level/census block group/neighborhood. Extensive research suggests that neighborhood income (median family income in the neighborhood) is a predictor of health [113,114,115,116].

#### 2.7.4. Confounders

*Age.* Parents reported the age of adolescents. Age was a continuous measure in years.

*Sex.* Sex was a dichotomous variable with males as 1 and females as 0.

*Marital Status.* Parental marital status was a dichotomous variable: married = 1, any other condition = 0.

*Race.* Race was self-identified and treated as a dichotomous variable: Black = 1, White = 0.

*Ethnicity.* Parents reported if they are of Hispanic ethnic background: Hispanic = 1 and = 0. Race/ethnicity was White = 0 and Black = 1.

*The Child Behavior Checklist (CBCL) Scores.* The Child Behavior Checklist (CBCL) also called the Achenbach System of Empirically Based Assessment (ASEBA) was used as a standard behavioral test to validate the left hippocampus function during the N-Back memory task. Using the CBCL, we measured the following eight sub-scores and also a total score: (1) anxious and depressed mood, (2) withdrawn and depressed affect, (3) somatic complaints, (4) social and interpersonal problems, (5) thought problems, (6) rule-breaking behaviors, (7) attention problems, (8) violent and aggressive behaviors, and CBCL total score [117]. The CBCL results predict the Diagnostic and Statistical Manual of Mental Disorders (DSM-IV-TR)-based psychiatric disorders [118]. The CBCL is based on parents’ reports and can screen the emotional health of adolescents. The CBCL is widely used across age groups, cultures, and settings and is well adapted to schools, medical settings, and mental health service delivery [95]. A literature review shows thousands of peer review publications using CBCL [95,96,97,98,99,100].

### 2.8. Data Analysis

To perform our multivariable analyses, we ran a hierarchical linear regression model. The independent variable was parental educational attainment. The outcome was left hippocampus function during the N-Back memory task. Control variables included race, age, sex, and marital status. Mediators were family income and neighborhood income. Unstandardized (b) regression coefficient, standard error (SE), confidence interval (CI), t, and p-values were reported for each model. A *p*-value of equal or less than 0.05 was significant. We also ran two series of bivariate correlations using Pearson correlation test. The first series of correlation were socioeconomic correlates. This helped us rule out multicollinearity between SES indicators and also establish the bivariate correlations required for the mediational hypothesis. This table was used to test if left hippocampus function during the N-Back memory task was correlated with parental education, family income, and neighborhood income. The second set of correlation matrix included left hippocampus function during the N-Back memory task and CBCL behavioral measures. This correlation matrix was used to establish the validity of left hippocampus function during the N-Back memory task, as hippocampus function is also supposed to be associated with social disfunction [119], behavioral problems [39], and aggression [43,119,120,121]. To perform our data analysis, we used SPSS (SPSS Inc, New York, USA).

## 3. Results

### 3.1. Descriptives

The current analysis was performed on 3067 8–11-year-old adolescents. From all participants, 2527 (71.2%) were non-Hispanic White, and 1023 (28.8%) were non-Hispanic Black. Table 1 presents the descriptive statistics of the sample.

### 3.2. Socioeconomic Correlates of Left Hippocampus Function during a Memory Task

Table 2 reports the results of bivariate correlations in the pooled sample. This model shows an association between marital status, family income, and parental educational attainment. This study also shows a correlation between marital status, family income, neighborhood income, and parental educational attainment with left hippocampus function.

### 3.3. Regressions

Table 3 reports the results of Model 1 in the pooled sample. This model was statistically significant (*p* < 0.05). In the absence of family and neighborhood income, parental educational attainment was associated with left hippocampus activation during the N-Back memory task (b = −0.04; *p* = 0.036). This effect was significant while age, sex, and marital status were controlled. Race was not associated with left hippocampus function during the N-Back memory task (b = 0.02; *p* = 0.338). Age (b = 0.00; *p* = 0.947), sex (b = 0.00; *p* = 0.949), and marital status (b = 0.01; *p* = 0.643) were not associated with hippocampus function during the N-Back memory task.

### 3.4. Mediation

Table 3 also shows the result of Model 2 that tested parental education mediation by family and neighborhood income. This model was statistically significant (*p* < 0.05). When we added SES indicators to our model (Model 2), the association between parental educational attainment and left hippocampus activation during the N-Back memory task was no more significant when we controlled for family income. Instead, a higher level of family income was associated with a lower level of left hippocampus activation during an N-Back memory task (b = −0.01; 95% CI = −0.02 to 0.00). This finding suggested that family income fully mediates (explains) the effect of parental educational attainment on left hippocampus activation during the N-Back memory task. Full mediation was defined and confirmed, according to Kenny [122]. The Sobel test was also used and confirmed a statistical mediation. These collectively suggested that mediation of the effect of parental educational attainment on left hippocampus activation during an N-Back memory task by family income is full. Neighborhood income, however, did not mediate the effect of parental education on outcome (*p* > 0.05).

### 3.5. Validation of the Hippocampus during N-Back Memory Task

As Table 4 shows that, activation of the left hippocampus during an N-Back memory task was positively correlated with CBCL total score as well as CBCL-social and interpersonal problems, CBCL-rule-breaking behaviors, and CBCL-violent and aggressive behaviors. As such, the left hippocampus during the N-Back memory task was correlated with some behavioral problems

## 4. Discussion

Four findings were observed. First, high family SES was associated with lower levels of hippocampus function during the N-Back memory task. Second, high parental education and family income but not neighborhood income are associated with lower levels of hippocampus function during the N-Back memory task. Third, high family income fully explains why high parental education is associated with lower hippocampus function during the N-Back memory task. Fourth, given that hippocampus function during the N-Back memory task was positively correlated with several aspects of the CBCL, parental education and family income were predictive of an fMRI market (hippocampus function during the N-Back memory task) which had some clinical significance in terms of social behaviors. The domains that our outcome correlated with included CBCL total score as well as CBCL-social and interpersonal problems, CBCL-rule-breaking behaviors, and CBCL-violent and aggressive behaviors.

There are a number of studies that have shown some related findings. A study by Barch et al. showed links between childhood poverty and reduced connectivity between the hippocampus and the amygdala and some other regions, including the superior frontal cortex, lingual gyrus, posterior cingulate, and putamen. The study showed that childhood poverty predicts connectivity between the left hippocampus and the right superior frontal cortex, and their brain connectivity mediate the effect of childhood poverty on adolescents’ depression [110].

Regarding our first and second findings, research shows that hippocampal function and size may be a function of education [123] and income [30]. This research has mainly linked smaller hippocampal sizes of to low SES and larger sizes of hippocampus in higher SES individuals’ hippocampal sizes [124,125,126].

This research suggests that a main reason hippocampal sizes follow SES is stress [124,127]. Stress-induced changes in hippocampal volume, particularly, the gray matter of the hippocampal is very well established and frequently seen in individuals with high chronic stress as well as poverty [128,129,130].

The effect of SES on the hippocampus may explain a wide range of behaviors and manifestations from memory to social behaviors and emotion regulation. Childhood poverty is linked to altered memory and hippocampal function [131]. Hippocampus, which has a role in emotion regulation, context processing, and memory has been also smaller in children of lower SES [30,64,132].

While our study focused on income as mediators of SES-hippocampus association, and while we studied the function rather than size of structure of the hippocampus, there are studies on the role of stress as explanatory mechanism between SES and hippocampal volume. In other words, low SES leads to an increase in chronic stress levels, and finally, this increase in stress levels results in structural and functional changes in the hippocampus. Several studies have established a link between SES and hippocampal volume [26,123]. Most of these studies have shown that low SES is associated with a lower size hippocampal [124,125,126]. Previous studies have found smaller hippocampal volume [30], and reduced memory recall paired with lower task-based hippocampal activation [30].

Our third finding can be explained through a theoretical lens as well. Mirowsky and Ross [85] and other investigators [91] proposed that income is one of the explanatory mechanisms by which education is linked to health. According to the scarcity hypothesis, low SES is a proxy of reduced availability and scarcity of resources necessary to protect adolescents against stress [133]. Low SES would reflect food and home insecurity, as well as economic difficulties that all can hinder adolescents brain development. In this view, a low level of access to resources is the underlying mechanism that explains why adolescents with low SES have poor brain development [133].

Low family SES (low income and parental education) is also a proxy of poor parenting [134,135,136,137,138] and high parental risk behaviors [139,140,141,142], which both can jeopardize the healthy brain development [143]. As a result of these cumulative risks, adolescents from low SES backgrounds become at an increased risk of psychopathology [144,145,146], problem behaviors [8,16,147], and poor school performance [148,149,150,151]. As low income is a better proxy of the scarcity of resources than parental education, income may better predict brain development than parental education.

As a side note, while parental education was controlled, race did not operate as a social determinant of hippocampus function during a memory task. We should emphasize that we conceptualized race as a social factor (a proxy of poverty and SES) rather than an innate, unchangeable biological factor. As race and SES did not show additive effects, low SES White and Black adolescents are at a high risk of hippocampus function during a memory task. As the altered activity of hippocampus is linked to a wide range of emotional, cognitive, and behavioral outcomes [36,152,153], the results have clinical and policy implication on how to reduce brain health inequalities and how to promote brain health equity across social groups.

Our study findings suggested that adolescents from families with low education are at risk because their low-income. That said, adolescents from families who have low income and uneducated parents may be at the highest risk for hippocampus activation during an N-Back memory task. Early childhood programs, after-school programs are among effective programs that have received some governmental attention [154,155,156,157]. Social policies should reduce the economic adversities of low-income families.

Although this study specifically focused on the altered activity of hippocampus, the hippocampus is not the only brain structure that is affected by SES. In a series of fMRI studies, Javanbakht et al. showed that low family SES impacts the amygdala response to negative face [2,68,69]. Another study by Yaple established hyperactivation of the reward network and hypoactivation of the executive network in low SES individuals [133]. Thus, the effects of SES, however, go beyond the hippocampus and can be seen for several other structures that regulate emotion regulation, memory, executive functioning, and cognition [158]. Some evidence suggests that the effects of poverty on some brain functions may be buffered by positive parenting [159]. Racial discrimination has shown to impact the hippocampus [160,161,162] and other brain regions such as the amygdala, putamen, the caudate, anterior insula, anterior cingulate, and medial frontal gyrus [163]. We, however, showed that SES, not race, determines hippocampus activity during a memory task.

Although low SES may alter the activity of the hippocampus, this study proposed that family income would be the final SES indicator that impacts hippocampus activity during a memory task. Using fMRI data from the ABCD study, we found that not parental education, but family income, is the main SES indicator that shapes hippocampus activation during a memory task.

Regarding our last finding, although hippocampus function is both correlated with memory, emotion, as well as behaviors. Hippocampus function correlates with CBCL-social and interpersonal problems, CBCL-rule-breaking behaviors, and CBCL-violent and aggressive behaviors. Although various brain regions and structures may carry some of the effects of low SES on brain function, the role of hippocampus is essential [24,25,26,27,28]. Hippocampus, a medial temporal lobe structure, the hippocampus, has major implications for a wide range of emotional, cognitive, and social behaviors including but not limited to memory [29,30,31,32,33], learning [34,35,36,37,38], behavioral maturation [164,165], emotion dysregulation [166,167], psychopthology [168], antisocial behaviors, behavioral problems, and conduct disorder [39], social disfunction [119], aggression [43,119,120,121], post-traumatic stress disorder (PTSD) [45,46,47,48,49,50,51], depression [52,53,54], and drug use [55,56,57,58,59]. Hippocampus is also showing a high degree of sensitivity to low SES [24,25,26,27,28]. Thus, altered hippocampus function may be one of the mediators of the effect of low family SES on poor adolescents’ learning and memory [12,25,27,35,60,61,62,63,64,65,66].

### 4.1. Implications

We found that family income fully explains why parental education impacts hippocampus function during a memory task. Given that family income is a modifiable factor through public and economic policies, manipulating income through minimum wage and taxation policies, the results advocate for income-redistribution policies to achieve brain health equality. Some example policies are earned tax income credit and minimum wage policies.

### 4.2. Limitations

To list some of the limitations, this study was cross-sectional in design. Thus, we cannot interpret the results as causal associations between SES and hippocampus function. Second, we only studied a limited number of SES indicators, and several neighborhood and contextual factors such as racial composition, segregation, air pollution, lead exposure, crime rate, or urbanity were not included. In addition, family income was a continuous measure with 10 non-evenly spaced bins. This can be also considered as another limitation of this paper. Despite these limitations, an advantage of this study was a large sample size and a national scope. Most previous studies on the link between SES and brain function have a lower number of sample sizes.

## 5. Conclusions

In summary, two SES indicators, namely parental education and family income, correlate with hippocampus activation during the N-Back memory task in a national sample of American adolescents. Family income, however, fully explains the effect of parental educational attainment on hippocampus activation during the N-Back memory task. This result provides additional evidence on how SES impacts adolescents’ brain development. As family income is responsible for the effects of parental education, we may be able to undo the effects of low education through taxation and minimum wage policies. In addition, given the effects of low SES, low-income adolescents may be disproportionately at risk. As income is a modifiable factor, and as income mediates the effect of parental education on adolescents’ brain development, policy solutions that alleviate poverty can contribute to the economic inequalities in adolescents brain development.

## Figures and Tables

**Table 1:** Descriptive data overall (n = 3067).

	*n*	%
**Race**		
White	2527	71.2
Black	1023	28.8
**Sex**		
Male	1712	48.2
Female	1838	51.8
**Marital status**		
Not-Married	1141	32.1
Married	2409	67.9
	**Mean**	**SD**
Age (Year)	9.45	0.51
Parental Education	16.87	2.44
Family Income	0.78	0.36
Neighborhood Median Income	7.37	2.43
Activation of the left hippocampus	–0.06	0.34

SD: standard deviation.

**Table 2:** Bivariate correlations (n = 3067).

	1	2	3	4	5	6	7	8
1 Race (Black)	1.00	0.02	0.02	−0.52 **	−0.42 **	−0.50 **	−0.52 **	0.06 **
2 Sex (Male)		1.00	0.03	0.01	0.00	0.00	0.01	0.00
3 Age (Year)			1.00	−0.02	−0.05 **	0.00	−0.02	−0.01
4 Family marital status (Maried)				1.00	0.41 **	0.40 **	0.58 **	−0.04 *
5 Parental educational attainment					1.00	0.50 **	0.62 **	−0.06 **
6 Family income						1.00	0.62 **	−0.05 **
7 Neighborhood income							1.00	−0.05 **
8 Left hippocampus function								1.00

* *p* < 0.05, ** *p* < 0.01.

**Table 3:** Linear regressions (n = 3067).

	Model 1 Main Effect							Model 2 Mediation						
	Beta	b	SE	95% CI		t	p	Beta	b	SE	95% CI		t	p
Race (Black)	0.02	0.01	0.02	−0.02	0.04	0.96	0.338	0.00	0.00	0.02	−0.03	0.03	−0.01	0.996
Sex (Male)	0.00	0.00	0.01	−0.02	0.02	−0.06	0.949	0.01	0.01	0.01	−0.02	0.03	0.56	0.574
Age	0.00	0.00	0.01	−0.02	0.02	−0.07	0.947	−0.01	0.00	0.01	−0.03	0.02	−0.31	0.759
Married	0.01	0.01	0.01	−0.02	0.04	0.46	0.643	0.02	0.01	0.02	−0.02	0.05	0.79	0.428
Parental education	−0.04	−0.01	0.00	−0.01	0.00	−2.10	0.036	−0.01	0.00	0.00	−0.01	0.01	−0.25	0.799
Family income	-	-	-	-	-	-	-	0.02	0.02	0.02	−0.02	0.06	1.00	0.318
Neighborhood income	-	-	-	-	-	-	-	−0.09	−0.01	0.00	−0.02	0.00	−2.93	0.003
Constant		0.03	0.12	−0.20	0.26	0.27	0.785		0.04	0.13	−0.20	0.29	0.34	0.731

Outcome: left hippocampus function. SE: standard error, CI: confidence interval.

**Table 4:** Validation of the activation of the left hippocampus during an N-Back memory task.

	1	2	3	4	5	6	7	8	9	10
1 Activation of the left hippocampus	1	0.00	0.01	−0.01	0.03 **	0.01	0.04 **	0.02	0.03 **	0.02 *
2 CBCL-Anxious and depressed mood (0–26)		1	0.58 **	0.47 **	0.62 **	0.60 **	0.41 **	0.57 **	0.58 **	0.77 **
3 CBCL-Withdrawn and depressed affect (0–14)			1	0.40 **	0.56 **	0.51 **	0.39 **	0.49 **	0.52 **	0.67 **
4 CBCL-Somatic complaints (0–16)				1	0.42 **	0.44 **	0.28 **	0.44 **	0.39 **	0.58 **
5 CBCL-Social and interpersonal problems (0–18)					1	0.62 **	0.55 **	0.69 **	0.67 **	0.82 **
6 CBCL-Thought problems (0–18)						1	0.51 **	0.73 **	0.63 **	0.81 **
7 CBCL-Rule-breaking behaviors (0–20)							1	0.65 **	0.74 **	0.73 **
8 CBCL-Attention problems (0–38)								1	0.76 **	0.90 **
9 CBCL-Violent and aggressive behaviors (0–38)									1	0.88 **
10 CBCL Total										1

CBCL: The Child Behavior Checklist; * *p* < 0.05, ** *p* < 0.01.

**Figure A1:**
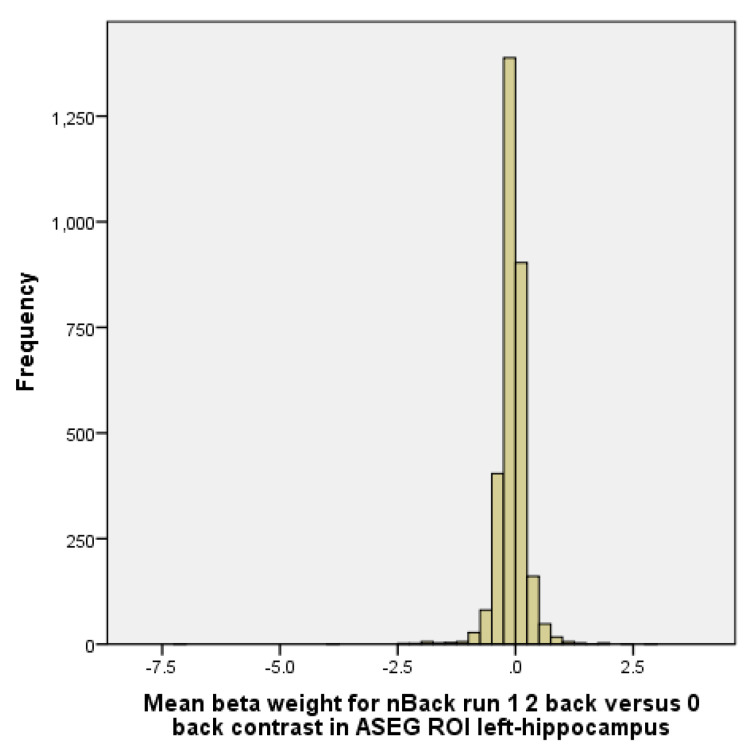
Histogram of hippocampus activation during the N-Back memory test.

## Appendix B

**Table A1:** Frequency of income in the sample.

Income Level	Assigned Code	n	%	Cumulative %
Less than $5000	1	111	3.6	3.6
$5000	2	126	4.1	7.7
$12,000	3	74	2.4	10.1
$16,000	4	114	3.7	13.8
$25,000	5	152	5.0	18.8
$35,000	6	224	7.3	26.1
$50,000	7	411	13.4	39.5
$75,000	8	436	14.2	53.7
$100,000	9	1059	34.5	88.2
$200,000	10	363	11.8	100.0
Total		3070	100.0	
